# Comparative study of sickle cell anemia and hemoglobin SC disease: clinical characterization, laboratory biomarkers and genetic profiles

**DOI:** 10.1186/s12878-017-0087-7

**Published:** 2017-09-15

**Authors:** Milena Magalhães Aleluia, Teresa Cristina Cardoso Fonseca, Regiana Quinto Souza, Fábia Idalina Neves, Caroline Conceição da Guarda, Rayra Pereira Santiago, Bruna Laís Almeida Cunha, Camylla Villas Boas Figueiredo, Sânzio Silva Santana, Silvana Sousa da Paz, Júnia Raquel Dutra Ferreira, Bruno Antônio Veloso Cerqueira, Marilda de Souza Gonçalves

**Affiliations:** 1 Laboratório de Hematologia e Genética Computacional, Instituto Gonçalo Moniz - IGM, Fundação Oswaldo Cruz (Fiocruz), Rua Waldemar Falcão, 121, Candeal, Salvador, Bahia CEP, 40296-710 Brazil; 2 Universidade Federal da Bahia (UFBA), Salvador, Bahia, Brazil; 3 Centro de Referência a Doença Falciforme, Itabuna, Bahia, Brazil; 4 Universidade Estadual de Santa Cruz (UESC), Ilhéus, Bahia, Brazil; 5 Universidade Estadual da Bahia (UNEB), Salvador, Bahia, Brazil

**Keywords:** Sickle cell anemia, Hemoglobin SC disease, Biomarkers, Genetic profile

## Abstract

**Background:**

In this study, we evaluate the association of different clinical profiles, laboratory and genetic biomarkers in patients with sickle cell anemia (SCA) and hemoglobin SC disease (HbSC) in attempt to characterize the sickle cell disease (SCD) genotypes.

**Methods:**

We conducted a cross-sectional study from 2013 to 2014 in 200 SCD individuals (141 with SCA; 59 with HbSC) and analyzed demographic data to characterize the study population. In addition, we determined the association of hematological, biochemical and genetic markers including the β^S^-globin gene haplotypes and the 3.7 Kb deletion of α-thalassemia (−α^3.7Kb^-thal), as well as the occurrence of clinical events in both SCD genotypes.

**Results:**

Laboratory parameters showed a hemolytic profile associated with endothelial dysfunction in SCA individuals; however, the HbSC genotype was more associated with increased blood viscosity and inflammatory conditions. The BEN haplotype was the most frequently observed and was associated with elevated fetal hemoglobin (HbF) and low S hemoglobin (HbS). The -α^3.7Kb^-thal prevalence was 0.09 (9%), and it was associated with elevated hemoglobin and hematocrit concentrations. Clinical events were more frequent in SCA patients.

**Conclusions:**

Our data emphasize the differences between SCA and HbSC patients based on laboratory parameters and the clinical and genetic profile of both genotypes.

## Background

Sickle cell disease (SCD) is a group of inherited diseases that includes sickle cell anemia (SCA), which is the homozygous state of the beta S (β^S^) allele and the most severe SCD genotype. Likewise, the heterozygous state of the β^S^ allele is characterized by the presence of hemoglobin S (HbS) associated with changes in the structure or synthesis of the other globin chain and consists of a group of less severe SCD, including hemoglobin SC disease (HbSC). SCD has important implications for public health, as both worldwide incidence and prevalence are high, which reinforces it as a significant social problem in many countries [1, 2]. The clinical diversity of SCD includes hemolytic and vaso-occlusive episodes (VOE), infections, stroke, acute chest syndrome (ACS), pulmonary hypertension, multiple organ dysfunctions and other complications [3]. Several factors have been shown to modulate the clinical manifestations of SCD including hematological, biochemical, inflammatory and genetic markers, as well as environmental, sociodemographic, and socioeconomic characteristics [3, 4].

With respect to the genetic markers, SCA patients can also be carriers of one or more gene determinants such as the 3.7 Kb deletion of α-globin chain in α-thalassemia (−α^3.7Kb^-thal). In Afro-descendants, the heterozygous (−α/αα) or homozygous (−α/−α) -α^3.7Kb^-thal genotype in SCA individuals is associated with a reduction in HbS concentration, which consequently lowers hemoglobin polymerization and cell damage and improves the hemolysis profile [5, 6]. In addition, this association promotes changes in hematological and biochemical parameters of SCA [5–7].

β^S^ globin gene haplotypes are constituted of polymorphisms in the β^S^ globin gene cluster, which are associated with specific levels of fetal hemoglobin (HbF), contributing to phenotypic diversity in SCA patients [8–10]. There are five main haplotypes, named Benin (BEN), Central African Republic (CAR), Senegal (SEN), Arab-Indian, and Cameroon (CAM), according to their geographical origin and ethnic groups [9, 11].

Considering the wide range of variability in the severity of SCA and HbSC individuals, laboratory biomarkers associated to hemolysis such as reticulocyte count and serum LDH, in addition to biomarkers of blood viscosity such as hemoglobin and hematocrit concentration, are important to perform a laboratorial characterization of the patients as well as to understand the disease physiopathology [12]. HbF levels, inflammatory response and endothelial dysfunction play a pivotal role in differentiating SCD sub-phenotypes [12, 13].

The severity of SCD arises from several clinical complications that influence each individual’s immunity. Therefore, the use of medication, prophylactic vaccines and practicing healthy habits are recommended [14, 15]. However, another important point is the cost to the health care system and how much the government pays for each patient with SCD, including hospital admissions and readmissions, therapy and the spectrum of comorbidities that may require years of follow-up in different specialists [15, 16].

In this study, we investigated the association of different clinical manifestations, laboratory biomarkers and genetic profiles in patients with SCA and HbSC to establish parameters that highlight sub-phenotypes differences in these SCD genotypes.

## Methods

### Subjects

We conducted a cross-sectional study from 2013 to 2014 at the Itabuna Reference Center for Sickle Cell Disease in Itabuna, Bahia, Brazil, that develop the follow-up of 536 SCD patients from the south coast, extreme south and southwest regions of Bahia. Considering the cross-sectional nature of the study, the sample size calculation was performed on StatCalc, Epi Info, v.6.04 with a power of 95% and two-sided confidence level of 95%. Thus, we identify that a sample-size of 200 individuals with SCD would be a significant representation of the population, taking account a frequency of 1/650 children with SCD in Bahia state. Our sample size was 200 SCD patients (141 with SCA and 59 with HbSC) with a mean age of 16.06 ± 11.83 years and a median age of 13 years (range: 1–61 years). Clinical data were collected from the medical records. Each patient enrolled in the study was in a steady state, had not received a blood transfusion in the last six months and were not taking hydroxyurea (HU). Informed consent form was obtained from all adult participants as well as parents or guardians of the children also have assigned the informed consent form prior to the enrollment in the study. The study protocol was approved by the Ethics Research Board of the Gonçalo Moniz Institute of the Oswaldo Cruz Foundation (IGM-FIOCRUZ-BA) following the ethical principles of the Declaration of Helsinki of 1975 and its revision.

### Hematological and biochemical parameters

Hematological analyses were carried out using Sysmex KX-21 N™ Automated Hematology Analyzer (Sysmex Corporation, Tokyo, Japan). Serum lipids and lipoproteins were analyzed using fully automated equipment (Cobas, Roche Diagnostics, Salt Lake City, Utah, USA). Hemoglobin profiles and HbF concentration were determined using High Performance Liquid Chromatography (HPLC, VARIANT I-Bio-Rad, CA, USA). Nitric oxide metabolites (NOm) were determined using the Griess reaction, as previously described [17].

### Genetic analysis

Genomic DNA was extracted from leukocytes using a QIAamp® DNA Extraction Kit (Qiagen, Hilden, Germany) following the manufacturer’s instructions. β^S^ globin gene cluster haplotypes were investigated using polymerase chain reaction (PCR) followed by restriction fragment length polymorphism (RFLP). The primers used detecting the β^S^ globin gene cluster haplotypes are: 5′γ^G^ gene 5′-AACTGTTGCTTTATAGGATTTT-3′ and 3′-AGGAGCTTATTGATAACTCAGAC-5′; γ^G^ /γ^A^ gene 5′-AAGTGTGGAGTGTGCACATGA-3′ and 3′-TGCTGCTAATGCTTCATTACAA-5′; γ^G^ /γ^A^ gene 5′- TGCTGCTAATGCTTCATTACAA-3′ and 3′-TAAATGAGGAGCATGCACACAC-5′; Ψβ gene 5′-GAACAGAAGTTGAGATAGAGA-3′ and 3′-ACTCAGTGGTCTTGTGGGCT-5′; 3′Ψβ gene 5′-TCTGCATTTGACTCTGTTAGC-3′ and 3′-GGACCCTAACTGATATAACTA-5′ [10, 18]. Allele-specific PCR was used to investigate the -α^3.7Kb^-thal deletion presence [19]. All analyses were performed in the Anemia Research Laboratory at the Federal University of Bahia and Laboratory of Hematology, Genetics and Computational Biology at the IGM-FIOCRUZ-BA.

### Statistical analysis

Baseline values of selected variables were summarized as the mean and stratified according to percentile. Distribution of the quantitative variables was analyzed using Shapiro-Wilk test. Quantitative variables were compared between two groups using the t-test for data with a parametric distribution and the Mann-Whitney test for nonparametric data. The Chi-square test and Fisher exact test were used to analyze the qualitative or categorical variables. Statistical analyses were performed using the Statistical Package for the Social Sciences (SPSS) version 20.0 software (IBM, New York, NY, USA), and *P* values <0.05 were considered significant.

## Results

In our study, we analyzed patients with SCA and HbSC and have identified that the majority of the SCD patients were female (52.0%; 104/200) and were aged either between 6 and 10 years (22.0%; 44/200) or 21 and 30 years (20.0%; 40/200). As recommended by the Brazilian Institute of Geography and Statistics the ethnicity was self-declared and the majority of patients were African derived people (89.0%; 178/200) (Table 1). With respect to patients’ educational level, the frequency of uneducated or partially completed elementary school was 12.0% (24/200) in the age group of 11 to 15 years old, and 8.5% (17/200) in the age group older than 16 years old. In addition, in the age group older than 16 years old we also observed a frequency of 20.0% (40/200) of the patients only completed elementary school (Table 1). The age at first diagnosis of SCD was younger than 6 months of age in the majority of the patients (38.0%; 76/200), and 68 patients in this group were diagnosed through the newborn screening. In addition, 25.0% of the patients were diagnosed between the age of 7 months and 4 years (50/200) and 17.0% were diagnosed between the ages of 5 and 9 years (34/200) (Table 1). When we analyzed the number of patients who have any relatives with SCD, we found that 31.5% (63/200) had a sibling with the disease, and 28.5% (57/200) had four or more sibling with SCD (Table 1).

**Table 1:** Characterization of SCD patients followed by the Reference Center in the South of Bahia, Brazil

Age (years)	N (%)	Sex	N (%)	Ethnicity	N (%)	Region of Origin		N (%)
≤ 5	36 (18.0)	Female	104 (52.0)	Caucasian	13 (6.5)	South Coast		156 (78.0)
6 to 10	44 (22.0)	Male	96 (48.0)	African	178 (89.0)	Extreme South		29 (14.5)
11 to 15	37 (18.5)			Asian	9 (4.5)	Southwest		15 (7.5)
16 to 20	21 (10.5)							
21 to 30	40 (20.0)							
≥ 31	22 (11.0)							
Number of siblings with SCD	N (%)	Age at 1st Diagnosis	N (%)	Relatives with SCD	N (%)	Education (by age group)		N (%)
0	21 (10.5)	≤ 6 months	76 (38.0)	None	100 (50.0)	≤ 5	Uneducated or incomplete	36 (18.0)
1	47 (23.5)	7 months to 4 years	50 (25.0)	Father	4 (2.0)		elementary school	
2	42 (21.0)	5 to 9 years	34 (17.0)	Mother	3 (1.5)	6 to 10	Uneducated or incomplete	34 (17.0)
3	33 (16.5)	10 to 14 years	11 (5.5)	Brother	63 (31.5)		elementary school	
4 or More	57 (28.5)	15 to 17 years	15 (7.5)	Cousin	22 (11.0)		Elementary school	10 (5.0)
		≥ 17 years	14 (7.0)	Aunt and Uncle	1 (0.5)	11 to 15	Uneducated or incomplete	24 (12.0)
				Nephews	2 (1.0)		elementary school	
				Not heard	5 (2.5)		Elementary school	13 (6.5)
				Inform		≥ 16	Uneducated or incomplete	17 (8.5)
							elementary school	
							Elementary school	25 (12.5)
							High School	40 (20.0)
							University	1 (0.5)

**Fig. 1:**
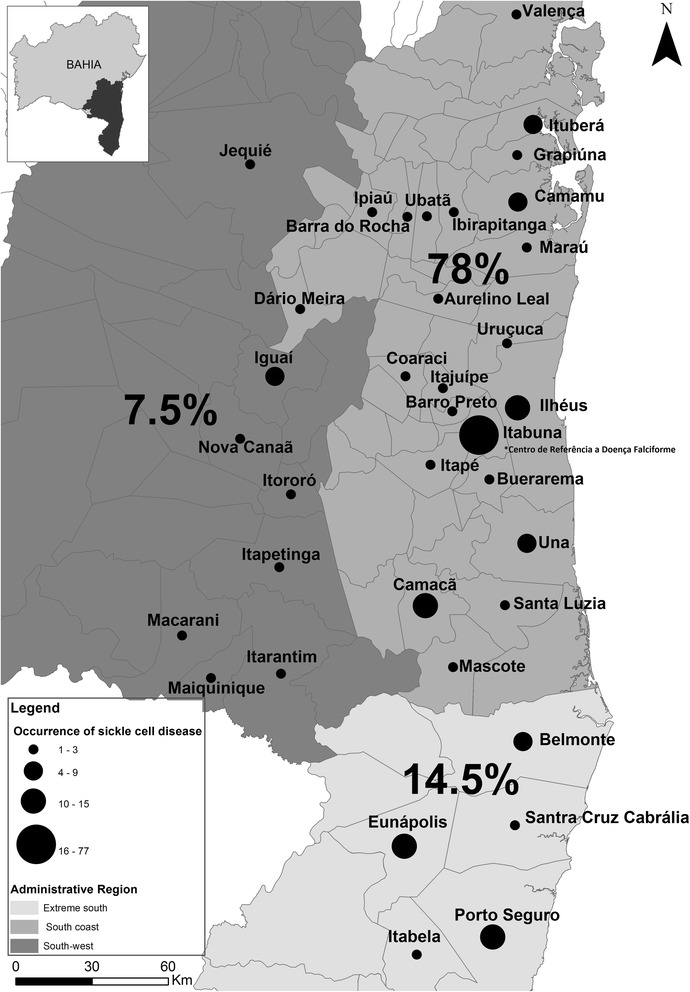
Distribution of SCD patients on South Coast, Extreme South and Southwest of Bahia

SCD patients were from several cities belonging to administrative regions, with 78.0% from the south coast of Bahia, 14.5% from the extreme south of Bahia, and 7.5% from the southwest of Bahia (Fig. 1). On the south coast, the city of Itabuna had the highest number of SCD patients, which represented 38.5% (77/200) of the study population, followed by Eunapólis, Ilhéus, Porto Seguro, and Camacan, which had 6.0% (12/200), 6.0% (12/200), 5.5% (11/200), and 5.0% (10/200) of the SCD patients respectively.

We found that BEN haplotype (47.9%; 135/282) was the predominant haplotype in SCA patients, followed by CAR haplotype (45.0%; 127/282), and atypical haplotype (7.1%; 20/282). In the HbSC patients, 54.2% had BEN haplotype (32/59) and 45.8% had CAR haplotype (27/59). Regarding the genotype of the β^S^ globin gene cluster haplotypes among the SCA patients, 41.2% (58/141) had BEN/CAR genotype, followed by CAR/CAR genotype (22.7%; 32/141) and BEN/BEN genotype (24.8%; 35/141). Among the SCA patients, we found that 5.0% had BEN/Atypical genotype (7/141), 3.5% had CAR/Atypical genotype (5/141), and 2.8% the atypical genotype (4/141). When we analyzed the β^C^ globin gene cluster haplotypes associated to the HbSC, we found that 5.1% (3/59) had the BEN II genotype, 35.6% (21/59) had the CAR I genotype, 47.4% (28/59) had the BEN I genotype, 8.5% (5/59) had the CAR III genotype, 1.7% (1/59) had the CAR II genotype, and 1.7% (1/59) had the BEN III genotype (Table 2). The haplotypes are known to modulate HbF levels, thus, we analyzed 99 patients with SCA and 52 patients with HbSC who had no history of HU therapy and absence of the CAR haplotype lead to elevated HbF levels (Table 3).

**Table 2:** Distribution of the genotypes and alleles of the β^S^ globin haplotype and -α^3.7Kb^-thalassemia in SCD

β^S^ globin Genotype				-α^3.7Kb^-Thalassemia Genotype			
HbSC	% (*N* = 59)	SCA	% (*N* = 141)	HbSC	% (N = 59)	SCA	% (N = 141)
BEN II	5.1 (3)	BEN/BEN	24.8 (35)	Absence	84.7 (50)	Absence	83.7 (118)
CAR I	35.6 (21)	BEN/CAR	41.2 (58)	Heterozygous	13.6 (8)	Heterozygous	14.2 (20)
BEN I	47.4 (28)	CAR/CAR	22.7 (32)	Homozygous	1.7 (1)	Homozygous	2.1 (3)
CAR III	8.5 (5)	BEN/Atypical	5.0 (7)				
CAR II	1.7 (1)	CAR/Atypical	3.5 (5)				
BEN III	1.7 (1)	Atypical	2.8 (4)				
β^S^ globin Haplotype in HbSC		β^S^ globin Haplotype in SCA		Frequency of -α^3.7Kb^-Thalassemia chromosome in SCD			
BEN	54.2 (32/59)	BEN	47.9 (135/282)	Presence 0.09 (36/400)			
CAR	45.8 (27/59)	CAR	45.0 (127/282)				
		Atypical	7.1 (20/282)

**Table 3:** Distribution of the median (25th – 75th) hematological data among the β^S^ globin haplotypes and -α^3.7Kb^-thalassemia

β^S^ globin Haplotype				-α^3.7Kb^-Thalassemia			
HbSC (*N* = 52)		SCA (*N* = 99)		HbSC (N = 52)		SCA (N = 99)	
Median (25th – 75th)	Median (25th – 75th)	Median (25th – 75th)	Median (25th – 75th)	Median (25th – 75th)	Median (25th – 75th)	Median (25th – 75th)	Median (25th – 75th)
Absence CAR *N* = 26	Presence CAR N = 26	Absence CAR *N* = 29	Presence CAR *N* = 70	Absence -α^3.7Kb^ *N* = 43	Presence -α^3.7Kb^ N = 9	Absence -α^3.7Kb^ *N* = 85	Presence -α^3.7Kb^ N = 14
**HbS**		**HbS**		**Hb**		**Hb**	
50.0 (48.1–51.4)	50.6 (49.5–51.6)	83.0 (76.0–88.3)	88.3 (83.5–91.7)	10.8 (10.1–11.6)	10.9 (9.9–12.4)	7.6 (7.1–8.3)	8.2 (7.3–8.8)
*p* = 0.301		***p*** **= 0.004**		*p* = 0.735		*p* = 0.427	
**HbF**		**HbF**		**Ht**		**Ht**	
1.9 (0.8–4.5)	1.3 (0.7–3.2)	12.9 (7.5–18.3)	7.7 (4.1–11.5)	30.7 (28.7–32.5)	31.7 (28.0–34.1)	21.7 (19.9–24.1)	23.8 (21.4–25.5)
*p* = 0.181		***p*** **= 0.005**		*p* = 0.961		*p* = 0.252	
				**MCV**		**MCV**	
				76.1 (71.5–80.1)	71.6 (67.3–72.4)	88.0 (83.6–93.2)	80.3 (76.3–83.1)
				***p*** **= 0.018**		**p < 0.001**	
				**MCH**		**MCH**	
				27.2 (24.6–28.8)	25.0 (23.4–26.5)	30.8 (28.8–32.7)	27.7 (25.7–29.0)
				*p* = 0.071		**p < 0.001**	

*Hb* hemoglobin, *Ht* hematocrit, *MCV* mean cell volume, *MCH* mean corpuscular hemoglobin. Bold values indicate significance at p < 0.05; p-value obtained using Mann-Whitney

We found that 168 patients had the wild type of the α- genotype and that 32 patients had the deletion (−α^3.7Kb^-thal). In this group, we observed 4 patients with the homozygous genotype (−α/−α) and 28 patients with the heterozygous genotype (−α/α) (Table 2). When we evaluated 98 patients with SCA and 52 patients with HbSC who had no history of HU therapy, we have found that hemoglobin and hematocrit concentrations were high and that mean cell volume (MCV) and mean corpuscular hemoglobin (MCH) were low in the presence of the α^3.7Kb^-thal in both SCD genotypes (Table 3).

Among the biomarkers of hemolysis, the red blood cell (RBC) count (*p* < 0.001), hemoglobin (p < 0.001) and hematocrit concentrations (p < 0.001) were low, and MCV (p < 0.001), MCH (p < 0.001) concentration and red blood cell distribution width (RDW) (p < 0.001) were high in SCA patients (Table 4). Additionally, in the same group, we found that the levels of total (p < 0.001), direct (p < 0.001) and indirect (p < 0.001) bilirubin, lactate dehydrogenase (LDH) (*p* < 0.001), NOm (*p* = 0.047) and reticulocyte count (p < 0.001) were high (Table 3). We also found that the total white blood cell (WBC) (p < 0.001), eosinophil (*p* = 0.007), lymphocyte (p < 0.001) and monocyte (*p* = 0.003) counts were high in SCA patients (Table 4). The platelet count was increased in SCA patients (p < 0.001). Conversely, we observed low levels of total cholesterol (*p* = 0.012) and high-density lipoprotein cholesterol (HDL-C) (p < 0.001) in these patients (Table 4).

**Table 4:** Comparison of the laboratory data between SCA and HbSC patients

Laboratory value	SCA (*N* = 98) Median (25th – 75th)	HbSC (N = 52) Median (25th – 75th)	p *value*
Hemolysis			
RBC, ×10^12^/L	2.60 (2.30–2.80)	4.15 (3.80–4.50)	**<0.001**
Hemoglobin, g/dL	7.60 (7.15–8.50)	10.90 (10.12–11.82)	**<0.001**
Hematocrit, %	21.80 (20.15–24.47)	30.80 (28.72–32.50)	**<0.001**
MCV, fL	86.05 (81.37–91.10)	74.40 (70.80–79.72)	**<0.001**
MCH, fL	30.15 (27.87–32.25)	26.60 (24.60–28.67)	**<0.001**
RDW, fL	24.80 (21.60–27.20)	18.65 (17.62–19.57)	**<0.001**
Total bilirubin, mg/dL	2.55 (1.67–3.72)	1.20 (0.80–1.67)	**<0.001**
Direct bilirubin, mg/dL	0.45 (0.30–0.60)	0.30 (0.20–0.40)	**<0.001**
Indirect bilirubin, mg/dL	2.20 (1.20–3.12)	0.90 (0.52–1.27)	**<0.001**
LDH, U/L	1094.00 (785.50–1684.50)	481.50 (381.75–567.25)	**<0.001**
Reticulocyte			
Reticulocyte count	5.40 (4.20–8.20)	3.65 (2.62–4.37)	**<0.001**
NO metabolites			
NOm, μM	35.62 (28.02–47.50)	31.34 (23.19–40.80)	**0.047**
Leukocytes			
WBC, × 10^9^/L	13.80 (11.17–16.10)	10.45 (7.22–12.90)	**<0.001**
Segment count, × 10^9^/L	5778.00 (4275.25–7203.50)	5182.00 (3498.50–6717.00)	0.080
Eosinophil count, × 10^9^/L	695.50 (281.50–1774.75)	421.50 (216.50–895.25)	**0.007**
Lymphocyte count, × 10^9^/L	5535.00 (4335.00–7156.50)	3356.50 (2492.00–4704.75)	**<0.001**
Monocyte count, × 10^9^/L	349.50 (219.00–670.75)	257.00 (144.50–402.75)	**0.003**
Platelets			
Platelet count, ×10^3^/mL	441.50 (365.25–547.00)	267.00 (189.50–389.00)	**<0.001**
MPV, fL	9.50 (8.80–10.40)	9.70 (9.30–10.40)	0.314
Lipid metabolism			
Total Cholesterol, mg/dL	122.00 (97.75–146.75)	133.50 (110.25–166.75)	**0.012**
HDL-C, mg/dL	32.00 (27.00–38.00)	37.00 (33.00–44.75)	**<0.001**
LDL-C, mg/dL	67.00 (41.75–88.25)	73.00 (57.00–109.75)	0.071
VLDL-C, mg/dL	22.00 (16.75–31.25)	21.00 (14.00–27.75)	0.178
Triglycerides, mg/dL	110.00 (82.75–156.50)	103.50 (68.50–138.50)	0.175

*RBC* red blood cells, *MCV* mean cell volume, *MCH* mean corpuscular hemoglobin, *RDW* red cell distribution width, *LDH* lactate dehydrogenase, *NOm*: nitric oxide metabolites, *WBC* white blood cell, *MPV* mean platelet volume, *HDL-C* high-density lipoprotein cholesterol, *LDL-C* low-density lipoprotein cholesterol, *VLDL-C* very low-density lipoprotein cholesterol; Bold values indicate significance at *p* < 0.05; *p*-value obtained using Mann-Whitney

We analyzed the distribution of clinical events in the different SCD genotypes (SCA and HbSC) with no history of HU therapy, and we found that hospitalization was strongly associated with the SCA genotype (91.8%; 90/98) (*p* = 0.001) (Table 5).

**Table 5:** Clinical characterization of SCA and HbSC patients

Clinical characterization	SCA N = 98	HbSC *N* = 52	*p value*
Hospitalization	90/98 (91.8%)	36/52 (69.2%)	**0.001**
Pneumonia	38/98 (38.8%)	22/52 (42.3%)	0.728
Splenomegaly	40/98 (40.8%)	18/52 (34.6%)	0.486
Asthma	6/98 (6.1%)	3/52 (5.8%)	1.000
Pain crises	83/98 (84.7%)	41/52 (78.8%)	0.374
Infection	36/98 (36.7%)	18/52 (34.6%)	0.859
Priapism	10/50 (20.0%)	5/22 (22.7%)	0.764
Vaso-occlusion	89/98 (90.8%)	42/52 (80.8%)	0.119
Retinopathy	3/98 (3.0%)	4/52 (7.7%)	0.236
Cholelithiasis	17/98 (17.3%)	4/52 (7.7%)	0.139

Comparison of clinical events among the SCA and HbSC genotypes calculated using the Fisher’s exact test. Bold values indicate significance at p < 0.05

With regard to the use of prophylactic penicillin, we identified that 87.5% (70/80) of SCD pediatric patients received prophylactic penicillin therapy. Following the use of prophylactic penicillin therapy, 92.5% (185/200) of SCD patients in all age groups reported the use of folic acid everyday (*p* = 0.046). HU therapy was used by 25.0% (50/200) of SCD patients all older than 6 years and was more frequently used in patients aged 11 to 31 years (p < 0.001).

## Discussion

The present study analyzed the laboratory, genetic, clinical and demographic characteristics of 200 SCD patients who were followed at the Reference Center living in the south of Bahia in the northeast of Brazil.

We observed low educational level in patients with SCD, which is in accordance with findings from studies in the United States and other Brazilian regions [20, 21]. Another study observed that sociodemographic characteristics had no influence on the development of SCD complications; however, age, low socioeconomic class and education level were associated with anemic crisis in SCD [22, 23].

Among our patients 38.0% were diagnosed younger than 6 months of age, and 25.0% were diagnosed between 7 months and 4 years old, which is consistent with a delayed diagnosis. Newborn screening for SCD started in 2001 in Brazil when the National Newborn Screening Program (PNTN)/Guthrie test was founded to test for hemoglobinopathies [24], establishing the important early diagnosis of SCD. Newborn screening for hemoglobinopathies resulted in a reduction of mortality and clinical complications in SCD patients in Brazil, due to the therapeutic and clinical monitoring of the child since the birth [24].

The evaluation of the geographic distribution of the SCD patients included in our study showed high occurrence of SCD patients on the south coast of Bahia, additionally, the majority of the patients were from the city of Itabuna. Itabuna was initially settled in 1867 by cowboys from Sergipe, when they started to immigrate to Vitória da Conquista [25]. Sergipean immigrants, from a state close to Bahia, initiated holdings on the Cachoeira River banks during the same period that the Jesuits provided a catechesis to the *Pataxó, Guerren* and *Camacã* natives on farms [25]. African derived people coming from the Sergipe and Bahia backlands in 1850 were attracted to the wealth of the region and the possibility of working on the cocoa farms [25]. In addition, slaves originated from different African tribes and African regions were brought from South Africa to the port of Ilhéus during the slave trade period.

Interestingly, some of the historical aspects suggest the possibility of greater dispersion of the β^S^ allele in Itabuna. The predominant haplotype in SCA patients was BEN/CAR, as well as in HbSC patients was the haplotype BEN I followed by CAR I. According to the literature, in 1678–1814, approximately 39 of the 1770 ships that exported tobacco from Bahia went to Congo and Angola, where they captured Africans for slave labor, which represents the genetic contribution from the central Atlantic region of Africa [26]. After 1815, Bahia was the only Brazilian state that restricted slave traffic through Ecuador, which explains the association between the genotypic frequencies in Bahia and Western Africa, principally the Benin region [27]. We found high levels of HbF and low levels of HbS in patients with the BEN haplotype. Most of the patients with the CAR haplotype had low HbF levels (below 5% of the total HbF), whereas carriers of the BEN haplotype had intermediate HbF levels (between 5 and 15%) and this is in agreement with a previous study [28].

In Brazil, the prevalence of -α^3.7Kb^-thal is associated with different ethnic groups that constitute the population [29]. We observed a high prevalence of -α^3.7Kb^-thal deletion in the studied population. It is estimated that in Brazil, the overall frequency of the α-thalassemia trait is between 1% and 3% [30]. According to our results, the association with co-inheritance of α^3.7Kb^-thal deletion lead to increased hemoglobin levels, despite the microcytosis, hypochromia and increased hematocrit levels. The literature reports that the homozygous and heterozygous states of -α^3.7Kb^-thal, are characterized by mild anemia, hypochromia and microcytosis. However, increased hematocrit levels are observed, which contribute to enhance blood viscosity, increase vaso-occlusion and the occurrence of clinical events [7, 31].

Several studies have demonstrated the pathophysiological mechanisms underlying SCA and HbSC. We observed that SCA patients had a more prominent hemolytic pattern compared to HbSC patients who presented a lower RBC count and hemoglobin concentration, and increased LDH levels. This is in agreement with previous studies that described that in HbSC patients the HbC presence induces the HbS polymerization; however, it occur in a reduced degree when compared to SCA patients. Thus, HbSC patients exhibits a less severe hemolytic anemia [2, 32].

We identified increased reticulocytes count in SCA patients in response to hemolysis. This is related to anemic stress that promotes the release of immature RBCs from the bone marrow, which consequently increases reticulocytes count on peripheral blood [33]. We observed a slight decrease in NOm in HbSC patients. The lysis of erythrocytes promotes the release of free Hb in the plasma, which promotes inflammatory and oxidative effects that contribute to endothelial dysfunction [12, 34]. Due to hemolysis, heme, reactive oxygen species (ROS) and arginase are released into the bloodstream, increasing oxidative stress and decreasing NOm levels [35, 36].

Patients with HbSC had lower leukocyte count than SCA patients. This result is consistent with previous findings that identify the same association [37]. Moreover, SCA patients had higher platelet counts; however, the mean platelet volume (MPV) was high in both the genotypes. An increase in platelet activation has been found in SCD patients during vaso-occlusive events. Platelets regulate hemostasis, but they are also responsible for inducing inflammation [38].

The analysis of the lipid profile in SCD patients have shown lower HDL-C levels, as well as values below the reference value, which represents an important cardiovascular risk factor [39–41]. Hypertriglyceridemia and increased very low-density lipoprotein cholesterol (VLDL-C) were observed in SCA patients in this study, although these results were not statistically significant. According to previous reports, SCD individuals have decreased lipid plasma levels during hemolytic stress and compared with normal individuals [42].

Clinical manifestations are considered a limiting factor that may influence patient mortality; these include pain crisis, VOE and the coexistence of comorbidities, such as legs ulcers, infection, cholelithiasis, splenomegaly, retinopathy, vascular necrosis, and neurological disorders, which have a negative effect on the cognitive development of these patients [11, 16]. As demonstrated by our results, we found increased hospitalizations in SCA patients, once this is the most severe genotype of SCD characterized by a higher incidence of clinical complications and hospitalizations than HbSC [16].

We found that a high percentage of patients used oral prophylactic penicillin, including Benzathine, which was administered in patients prior to age 7 years. The prophylactic use of penicillin significantly reduces the risk of sepsis and death due to pneumococcal infection [43]. Another prescribed drug, folic acid, is used in cases of increased activity secondary to chronic hemolysis that results in high RBC destruction and leads to a deficit in folic acid [43, 44]. Our data showed daily high adherence to folic acid use in all age groups [45].

## Conclusion

Our data highlight the differences between sub-phenotypes among SCA and HbSC patients, based on laboratory characterization, genetic profiles and clinical manifestations of both genotypes. The results of our analyses emphasize the need for specialized care services for SCA and HbSC patients, particularly because of their heterogeneous genetic, clinical and pathophysiological backgrounds, and indicate the need for public health policies that significantly improve the health of these patients.

## Abbreviations

ACS: Acute chest syndrome
BEN: Benin
CAM: Arab-Indian, and Cameroon
CAR: Central African Republic
HbC: Hemoglobin C
HbF: Fetal hemoglobin
HbS: Hemoglobin S
HbSC: Hemoglobin SC disease
HDL-C: High-density lipoprotein cholesterol
HU: Hydroxyurea
LDH: Lactate dehydrogenase
MCH: Mean corpuscular hemoglobin
MCV: Mean cell volume
MPV: Mean platelet volume
NOm: Nitric oxide metabolites
PCR: Reaction polimerase chain
RBC: Red blood cell
RDW: Red blood cell distribution width
RFLP: Restriction fragment length polymorphism
ROS: Reactive oxygen species
SCA: Sickle cell anemia
SCD: Sickle cell disease
SEN: Senegal
VLDL-C: Very low-density lipoprotein cholesterol
VOE: Vaso occlusion events
WBC: White blood cell
-α^3.7Kb^-thal: -α^3.7Kb^-thalassemia
